# Maternal, prenatal and perinatal characteristics and first trimester maternal serum hormone concentrations

**DOI:** 10.1038/sj.bjc.6604639

**Published:** 2008-09-02

**Authors:** R Troisi, R N Hoover, R Thadhani, C-C Hsieh, P Sluss, R Ballard-Barbash, N Potischman

**Affiliations:** 1 Epidemiology and Biostatistics Program, Division of Cancer Epidemiology and Genetics, 6120 Executive Boulevard, Rockville, MD 20892-7246, USA; 2 Massachusetts General Hospital, 55 Fruit Street, Boston, MA 02114, USA; 3 University of Massachusetts Medical School, 373 Plantation Street, Worcester, MA 01605, USA; 4 Applied Research Program, Division of Cancer Control and Population Sciences, National Cancer Institute, 6130 Executive Boulevard, Rockville, MD 20892-7344, USA

**Keywords:** hormones, birth weight, maternal age, parity, smoking

## Abstract

In uncomplicated pregnancies, first trimester androgen, oestrogen and prolactin concentrations were higher in nulliparous (*n*=160) than parous (*n*=260) mothers. Androgens and estrogens were higher in younger than older mothers. These data are consistent with elevated hormone concentrations mediating the breast cancer protection from a first pregnancy and pregnancies occurring at younger ages.

There are few opportunities to directly evaluate the hypothesis that the pregnancy hormonal milieu influences cancer risk in the mother and offspring. Investigations have instead used proxies to reflect the endocrine environment such as birth weight which is associated with a modestly elevated breast cancer risk in the daughter (Michels and Xue, 2006). Whereas age at first and last pregnancy and parity are established maternal breast cancer risk factors (National Cancer Institute, 2003), associations have been less consistent for other pregnancy characteristics with regard to the mother (Cnattingius *et al*, 2005) and offspring (Potischman *et al*, 2004).

The few studies on hormone variations by maternal and pregnancy characteristics have focused on maternal measures in mid- or late pregnancy, primarily estrogens. Because the timing of carcinogenic events is unknown, characterising hormones over the entire pregnancy may provide additional insights into the biological mechanisms for maternal and perinatal breast cancer risk factors.

## Materials and methods

Data derived from an obstetrical study at the Massachusetts General Hospital (MGH) in Boston, Massachusetts that began in 1998 (Thadhani *et al*, 2001). Women were recruited at their first prenatal visit (ranging from 6.4 to 19.9 weeks gestation (median 11) with over 90% in the first 13 weeks), interviewed, and asked to provide a blood sample. Participants gave informed consent and the study was approved by the MGH Institutional Review Board.

The study sample, described previously (Potischman *et al*, 2005), includes 109 Hispanic, 56 African American and 255 Caucasian women 18–42 years of age (median 29.6), who delivered a live, singleton birth in an uncomplicated pregnancy. A total of 38% of the women were nulliparous.

### Laboratory assays

Sample handling, storage and assays were described previously (Potischman *et al*, 2005). Intra-class correlation coefficients from repeated measures of blind quality control samples were 97% for oestradiol, 92% for SHBG, 90% for oestrone, 85% for androstenedione, 76% for prolactin, 76% for testosterone, 60% for progesterone and <50% for DHEAS. Hormone medians and ranges have been published (Potischman *et al*, 2005).

### Statistical methods

Spearman correlation coefficients were calculated using continuous data. All pregnancy factors associated with the hormones in unadjusted analyses were entered with gestational age at blood collection (excluding pregnancies with 14+ weeks' gestation) into linear regression models with logarithm-transformed hormones as dependent variables. Exponentiated *β* estimates (−1.0) from these models are presented for factors which remained significantly associated with the hormones, or changed <10% from the unadjusted models. Interactions between maternal age (<30, 30+ years) and parity (nulliparous, parous) were tested by including the main effects and cross-product terms in a regression model. Additional interactions between all pregnancy factors and hormones by offspring sex and race were evaluated. Statistical significance was defined as *P*<0.05.

## Results

Gestational age at blood collection was inversely correlated with the androgens and positively correlated with the estrogens, progesterone, prolactin and SHBG (Table 1). Correlations with first trimester hormones were demonstrated for maternal age, weight and BMI, but not maternal height or birth weight.

In analyses mutually adjusted for the pregnancy factors and including gestational age, androgen and oestrogen concentrations were lower in older mothers (Table 2); the greatest incremental difference was between those <30 and 30+ years (e.g., *P*<0.001 for androstenedione; other data not shown). In contrast, maternal age was positively associated with progesterone. The androgens, estrogens and prolactin were higher in the 160 nulliparous than in the 260 parous women. Maternal BMI (and maternal weight; data not shown) was positively associated with the androgens and inversely associated with progesterone and SHBG, and maternal height was inversely associated with the estrogens and SHBG though not with the other hormones. Current smokers had lower progesterone and prolactin concentrations, whereas alcohol users had lower oestradiol only.

The positive association of maternal BMI and androstenedione was limited to mothers of female fetuses (*P*=0.01); there were no other interactions by offspring sex. Differences in hormones by race/ethnicity were reported in an earlier paper from this study (Potischman *et al*, 2005). The associations between the pregnancy factors and hormones were similar with adjustment for race/ethnicity and there were no meaningful interactions demonstrated (data not shown).

Except for progesterone, the highest hormone concentrations were generally observed in nulliparous women less than age 30 years and the lowest concentrations in parous women age 30 years and over although none of the interactions were significant (*P* ranged from 0.36 for SHBG to 0.79 for prolactin). In contrast, nulliparous women age 30+ years had higher progesterone concentrations than younger nulliparous women or parous women of any age (*P* for interaction=0.006).

## Discussion

There are few previous data characterising hormone variations in the first trimester. Our results for first compared with subsequent full-term pregnancies confirm previous findings of higher oestradiol (Bernstein *et al*, 1986), while also demonstrating elevations in androgens and prolactin. We had limited power to assess reported oestrogen differences by smoking status (Bernstein *et al*, 1989), thus the lower progesterone and prolactin concentrations we observed among smokers may be noteworthy.

Birth weight, an established prenatal breast cancer risk factor (Michels and Xue, 2006), is hypothesised to be mediated by foetal oestrogen exposure (Michels *et al*, 1996). Third trimester maternal oestriol is elevated in high birth weight pregnancies (Kaijser *et al*, 2000; Mucci *et al*, 2003; Peck *et al*, 2003; Troisi *et al*, 2003; Nagata *et al*, 2006), but data for second trimester estrogens are conflicting (Kaijser *et al*, 2000; Wuu *et al*, 2002). Our data show no association of birth weight with first trimester estrogens seeming to suggest that if oestrogen explains the association of birth weight and breast cancer risk, the critical exposure window may be later in pregnancy. Yet associations of birth weight with cord estrogens are unclear (Simmons *et al*, 1994; Shibata *et al*, 2002; Troisi *et al*, 2003; Nagata *et al*, 2006), and breast cancer risk in women prenatally exposed to diethylstilbestrol does not differ by trimester of first exposure (Palmer *et al*, 2006). Other prenatal factors could act differently. For example, a more pronounced breast cancer risk is suggested for women who were *in utero* during the Dutch famine in early compared with later gestation (Painter *et al*, 2006).

Long-term maternal breast cancer risk is reduced with a full-term pregnancy, particularly when it occurs at a young age (National Cancer Institute, 2003). Epidemiologic data indicate protection increases with gestational length (Vatten *et al*, 2002), suggesting that the important hormonal events must occur late in pregnancy. However, protection from a full-term pregnancy would also be consistent with cumulative dose effects of specific hormones throughout the pregnancy, or early hormonal events which require the subsequent action of others to induce the relevant molecular changes. Descriptive data characterising the hormonal profile of the entire pregnancy are lacking with regard to parity. The elevated first trimester oestradiol we observed in first compared with subsequent pregnancies has also been shown for the second (Wuu *et al*, 2002; Arslan *et al*, 2006) and early third trimesters (Wuu *et al*, 2002), but perhaps not at delivery (Troisi *et al*, 2003). The elevated prolactin concentrations in our data for first pregnancies have also been noted in the second trimester (Xu *et al*, 2003; Arslan *et al*, 2006), but not later in the pregnancy (Xu *et al*, 2003), and the first trimester androgen elevations have not been shown at delivery (Troisi *et al*, 2003). Our data also indicate that oestrogen and androgen concentrations are higher in pregnancies that occur in younger than in older women. There is limited and conflicting information for maternal estrogens by age at any point during the pregnancy (Panagiotopoulou *et al*, 1990; Kaijser *et al*, 2002; Troisi *et al*, 2003), whereas the androgen findings are consistent with studies using measurements made later in the pregnancy (Carlsen *et al*, 2003; Troisi *et al*, 2003).

The explanation for the protective effect of a first pregnancy and the inverse relationship of this protection with the age at which the pregnancy occurs remains speculative. The hypothesis most commonly invoked is that irreversible molecular changes (driven by hormonal exposures) to the breast that prevent tumour initiation (Medina, 2005) must occur early in life to precede the initial stage of breast carcinogenesis, and that most of these changes occur with the first pregnancy. An alternative explanation, however, is that the operative hormonal influences may be different in first pregnancies, particularly those occurring at young ages. Our finding of higher circulating estrogens, androgens and prolactin in both a first pregnancy and, independently, in those occurring at younger ages could be consistent with this latter explanation. Subsequently reduced concentrations of these hormones in the pregnant and non-pregnant state may also be involved.

The study subjects were participants in a health care plan that provides prenatal care. The MGH study's participation rate is high and thus unlikely to represent an unusual population of women who seek medical care early in pregnancy. Whereas the reproducibility data were high for oestradiol and oestrone, there was substantial laboratory imprecision for other analytes that based on the quality control data suggested sporadic, random measurement errors. Finally, it is unknown how well a single first trimester hormone measurement represents early pregnancy exposure.

Our data suggest that birth weight is not a marker of early pregnancy hormonal exposure, and that if hormones mediate any of the birth weight and breast cancer association the effect is likely to occur later in the pregnancy. Elevated first trimester oestrogen, androgen and prolactin concentrations in first full-term pregnancies, and elevated oestrogen, androgen and progesterone concentrations in pregnancies occurring at younger ages may be consistent with these hormones acting to reduce later breast cancer risk.

## Figures and Tables

**Table 1:** Spearman correlations between maternal hormone concentrations and maternal, gestational and neonatal characteristics (*n*=420)

	**Androstenedione**	**Testosterone**	**DHEAS**	**Estradiol**	**Estrone**	**Progesterone**	**SHBG**	**Prolactin**
Gestational age^a^	−0.15^**^	−0.15^**^	−0.21^***^	0.42^***^	0.31^***^	0.31^***^	0.45^***^	0.17^***^
Maternal age	−0.30^***^	−0.20^***^	−0.34^***^	−0.26^***^	−0.19^***^	0.09	−0.03	−0.17^***^
Maternal pregnancy weight^a^	0.14^**^	0.31^***^	0.14^**^	−0.02	−0.03	−0.27^***^	−0.27^***^	−0.01
Maternal height	0.03	0.05	0.06	−0.09	−0.08	0.00	−0.09	−0.05
Maternal BMI^a^	0.12^**^	0.30^***^	0.12^*^	0.02	0.02	−0.31^***^	−0.25^***^	0.02
Birth weight	−0.01	0.02	−0.01	0.05	0.04	−0.02	0.01	−0.06

^*^P<0.05; ^**^P<0.01; ^***^P<0.001.

a At blood draw (first prenatal visit).

**Table 2:** Results of linear regression models predicting maternal hormone concentrations^a^

	**Percent change (*P*-value)**							
**Independent variables**	**Androstenedione**	**Testosterone**	**DHEAS**	**Estradiol**	**Estrone**	**Progesterone**	**SHBG**	**Prolactin**
Maternal age (years)	−2% (<0.0001)	−2% (0.0005)	−3% (<0.0001)	−2% (0.0001)	−2% (0.01)	1% (0.12)		
Nulliparous *vs* parous		19% (0.002)	23% (0.0008)	17% (0.002)	24% (0.003)			59% (<0.0001)
Maternal height (cm)				−0.6% (0.04)	−0.9% (0.04)		−0.7% (0.0003)	
Maternal BMI at initial visit (kg m^−2^)	1% (0.004)	3% (<0.0001)	2% (0.0004)			−2% (<0.0001)	−2% (<0.0001)	
Current smoker *vs* past/never						−21% (0.001)		−19% (0.03)
Ever alcohol intake *vs* never				−11% (0.02)				
Education (<13 *vs* 13+ years)								

a Estimates are from regression models including all factors that were associated with the hormones in the categorical analyses and gestational age at blood collection and are presented if they remained statistically significant or if they changed less than 10% in the full model. The estimates represent the percent increase or decrease in the hormone concentration for each unit increase in the independent variable. Analyses excluded (*n*=10) subjects with gestational lengths ⩾14 weeks to restrict the findings to the first trimester; *n*=410.
